# Molecular Basis of Medullary Thyroid Carcinoma: The Role of *RET* Polymorphisms

**DOI:** 10.3390/ijms13010221

**Published:** 2011-12-27

**Authors:** Lucieli Ceolin, Débora R. Siqueira, Mírian Romitti, Carla V. Ferreira, Ana Luiza Maia

**Affiliations:** Thyroid Section, Endocrine Division, Hospital de Clínicas de Porto Alegre, Universidade Federal do Rio Grande do Sul, Rua Ramiro Barcelos 2350, 90035–003, Porto Alegre, RS, Brazil; E-Mails: lu.ceolin@gmail.com (L.C.); deborarsiqueira@yahoo.com.br (D.R.S.); mirianromitti@bol.com.br (M.R.); carlinhavaz@yahoo.com.br (C.V.F.)

**Keywords:** medullary thyroid carcinoma, *RET* polymorphisms, prognosis

## Abstract

Medullary thyroid carcinoma is a rare malignant tumor originating in parafollicular C cells. It accounts for 5 to 8% of all thyroid cancers. MTC develops in either sporadic (75%) or hereditary form (25%). Genetic and molecular studies have demonstrated the involvement of the *RET* proto-oncogene in hereditary MTC and, less often, in its sporadic form. Although a strong genotype-phenotype correlation has been described, wide clinical heterogeneity is observed among families with the same *RET* mutation or even in carriers of the same kindred. In recent years, several single nucleotide polymorphisms of the *RET* gene have been described in the general population as well as in patients with MTC. Some studies have reported associations between the presence of polymorphisms and development or progression of MTC. Nonetheless, other studies failed to demonstrate any effect of the *RET* variants. Differences in the genetic background of distinct populations or methodological approaches have been suggested as potential reasons for the conflicting results. Here, we review current knowledge concerning the molecular pathogenesis of sporadic and hereditary MTC. In particular, we analyze the role of *RET* polymorphisms in the clinical presentation and prognosis of MTC based on the current literature.

## 1. Molecular Basis of Medullary Thyroid Carcinoma

Medullary thyroid carcinoma (MTC) is a rare malignant tumor originating in parafollicular C cells of the thyroid first described by Hazard *et al*. [1]. MTC accounts for 5 to 8% of all thyroid gland tumors and its main secretory product is calcitonin. MTC may occur sporadically, in approximately 75% of cases, or as part of the inherited cancer syndrome known as multiple endocrine neoplasia type 2 (MEN 2) [2–4]. The reported 10-year mortality rate for patients with MTC varies from 13.5 to 38% [5–7].

The hereditary form of MTC is associated with germline mutations in the *RET* (*RE arranged during Transfection*) proto-oncogene, and presents as an autosomal dominant disease with a high penetrance and variable phenotype. *RET* point mutations are described mainly in exons 10, 11 and 16. However, less frequent mutations also occur in exons 5, 8, 13, 14 and 15 [8–13]. Hereditary MTC, also referred to as MEN 2, may be classified into three clinically distinct forms: multiple endocrine neoplasia type 2A (MEN 2A), type 2B (MEN 2B) and familial medullary thyroid carcinoma (FMTC) [11,12].

The molecular mechanisms involved in the sporadic MTC have not yet been clarified. About 50–80% of the cases present a somatic *RET* mutation M918T (Met/ATG → Thr/ACG, exon 16) [14–17]. However, the mutation does not appear to be uniform among the various cell subpopulations in the tumor or in the metastases, suggesting that sporadic MTC might be of polyclonal origin, or that the mutations in the *RET* proto-oncogene are not initial events in MTC tumorigenesis [14,16].

This review aims at presenting an updated picture of the current knowledge on the molecular pathogenesis of sporadic and hereditary MTC. In particular, we critically analyze the role of *RET* polymorphisms in the clinical presentation and prognosis of MTC.

## 2. The *RET* Proto-Oncogene

Genetic and molecular studies have shown the contribution of the *RET* proto-oncogene in hereditary MTC and, less often, in its sporadic form. The *RET* gene was identified in 1985 by Takahashi *et al*. during a classical experiment of NIH 3T3 cell transfection with the high molecular weight DNA of human T-cell lymphoma, hence the naming of the gene as *RET* (*RE arranged during Transfection*) [18]. Later, studies determined the *RET* location in chromosome 10 and related it to the genesis of MEN 2A, MEN 2B and FMTC [19,20]. In 1993, for the first time, point mutations in the *RET* gene were described in patients with MEN 2A and FMTC [9,13] and in the subsequent year, a specific *RET* mutation (M918T) was associated with MEN 2B and sporadic MTC [21].

**Figure 1:**
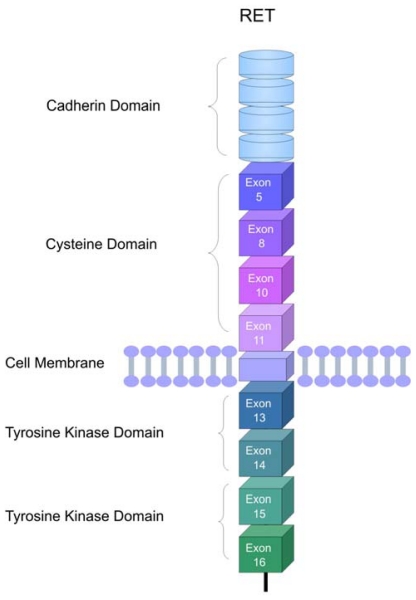
Schematic representation of the *RET* receptor. The extracellular region comprises the cadherin and cysteine rich domain. A single transmembrane region spans the cell membrane. Two tyrosine kinase domains (TK1 and TK2) are located in the intracellular region. The corresponding exons coding for the cysteine and thyrosine kinase domains are indicated.

The *RET* gene encodes a receptor tyrosine-kinase, expressed in the cells derived from the neural crest: thyroid parathyroid cells (C cells), chromafin cells of the adrenal medulla and enteric autonomic plexus. Since it is a membrane receptor, the RET protein is constituted by three domains: an extracellular domain, a transmembrane domain and an intracellular portion containing two tyrosine-kinase domains (Figure 1). The extracellular domain includes regions homologous to the cadherin family of cell adhesion molecules and a large region rich in cysteine residues that performs the transduction of extracellular signals of proliferation, growth, differentiation, migration, survival and cell apoptosis. The intracellular domain is divided into 2 tyrosine-kinase subdomains (TK1 and TK2), separated by 28 aminoacids. These subdomains contain the tyrosine residues that are phosphorylated during receptor activation, and are involved in the activation of the signaling intracellular pathways. *RET* is subject to alternative splicing of the 3′ region generating three protein isoforms that contain 9 (*RET*9), 43 (*RET*43) and 51 (*RET*51) amino acids in the carboxy-terminal tail downstream from glycine 1063. *RET*9 and *RET*51, consisting of 1072 and 1114 amino acids, respectively, are the main isoforms *in vivo* [22,23].

## 3. RET Protein Activation

The RET receptor tyrosine kinase is activated through a complex formed by the glial cell line-derived neurotrophic factor (GDNF) family of ligands and co-receptors. Under normal conditions, RET activation depends on the interaction of GFRαs (GDNF Family α Receptor) co-receptors and their respective ligands GFLs (GDNF Family of Ligands). The GFRα-ligand complex, together with the extracellular portion of RET, promotes autophosphorylation of the intracellular tyrosine residues [24,25].

**Figure 2:**
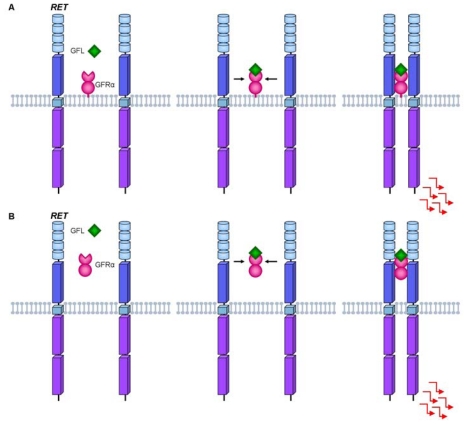
Mechanisms of ligand-mediated RET activation. (**A**) In the *cis* model RET activation: the glial cell line-derived neurotrophic factor (GDNF) family of ligands (GFL) binds to membrane glycosylphosphatidylinositol-anchored GDNF-family coreceptors (GFRα). The activation leads to dimerization of RET and consequently activation of the intracellular signaling pathways; (**B**) In the *trans* model RET activation: the ligand binds to the soluble form of its coreceptor (GFRα) and the ligand-GFRα complex brings together two inactive RET monomers. Ligand-induced activation induces dimerization and tyrosine phosphorylation of the RET receptor with downstream activation of several signal transduction pathways.

The RET co-receptors are usually bound to the plasma membrane, but GFRs also occur in a soluble form, and can then activate RET in two distinct forms: *cis* or *trans* (Figure 2). The *cis* model for the RET activation hypothesis occurs when the GFL ligand binds to the GFRα co-receptor anchored on a lipid platform and later this complex promotes the approach of two RET molecules through the lipid platform, allowing the phosphorylation of the intracellular tyrosine residues. On the other hand, the *trans* model activation suggests that the GFL may also bind to the soluble form of GFRα, stimulating the dimerization of RET outside the lipid platform, thus allowing its activation. Once activated, RET initiates the different intracellular pathways involving the regulation of processes such as differentiation, survival, proliferation, migration and cell chemotaxis [24,26].

**Figure 3:**
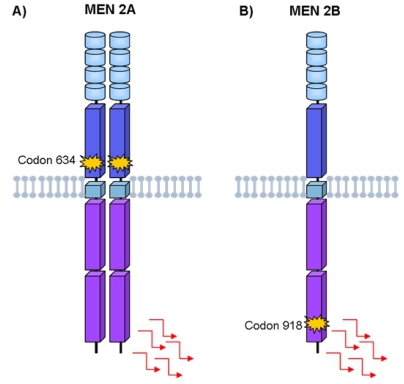
Characterization of *RET* oncogenic activation in MEN2 inherited cancer syndromes. (**A**) MEN 2A *RET* mutation leaves an unpaired cysteine residue in a RET monomer to form an aberrant intermolecular disulfide bond with another mutated monomer. The two mutated RET molecules are constitutively dimerized and activated; (**B**) MEN 2B *RET* mutation activates tyrosines in the kinase domain and alters its substrate specificity leading to aberrant phosphorylation of substrates of RET receptor.

The molecular mechanism by which *RET* mutations trigger the neoplastic process was determined by elegant *in vitro* studies performed by Santoro *et al.* [27]. Briefly, under normal conditions, RET is only activated in the presence of GFRα/GFL complex, which on binding to the RET receptor promotes its dimerization and auto-phosphorylation of the intracellular signaling pathways. The presence of mutation in the extracellular domain, as found in MEN 2A, leads to the dimerization of RET even in the absence of the ligand, with consequent constitutive activation of the intracellular signaling pathways (Figure 3A). Mutations in the intracellular tyrosine-kinase domain, as found in MEN 2B, alter RET substrate specificity due to structural changes in this domain. Consequently, the mutated RET no longer needs dimerization to become active (Figure 3B) [28,29]. The activation of the RET protein appears to be an initial step in the oncogenic pathway in the tissues where it is expressed. Molecular evidence of other chromosomal abnormalities, such as loss of heterozygosity, most often at 1p and 22q, suggest that additional cytogenetic events are probably involved [11,30].

## 4. Hereditary Medullary Thyroid Carcinoma

Approximately 25% of MTC cases occur as part of the inherited cancer syndrome of MEN 2 [7,31]. The MEN 2A subtype constitutes approximately 70%–80% of cases of MEN 2 and is characterized by the presence of MTC (95%), pheochromocytoma (30–50%) and hyperparathyroidism (HPT) (10–20%). The MEN 2B syndrome accounts for about 5% of the cases of MEN 2. The frequency of MTC is over 90%, pheochromocytoma (45%), ganglioneuromatosis (100%) and marfanoid habitus (65%) [11,32]. This syndrome is characterized by a single phenotype, which includes diffuse ganglioneuromatosis of the tongue, lips, eyes and gastrointestinal tract, long fingers and extremities, hyperextension of the joints and epiphyseal abnormalities. MTC in the setting of MEN 2B develops earlier and has a more aggressive course, occurring at a younger age compared with MTC in other MEN 2 subtypes [6,7]. The FMTC subtype constitutes approximately 10 to 20% of the cases of MEN 2 [11]. MTC is the only manifestation and thereby it is necessary to demonstrate the absence of a pheochromocytoma or hyperparathyroidism in two or more generations of the same family or the identification of related mutations to confirm that particular kindred have this syndrome. In these cases, the clinical presentation of MTC occurs later and the prognosis is more favorable (corresponding to older age at onset, often between 20 and 40 years) compared to the other forms of MTC [33].

### Germline *RET* Mutations and Disease Phenotype

Several studies indicate a correlation among specific *RET* mutations (genotype) and age of onset, aggressiveness of MTC and the presence or absence of other endocrine neoplasms (phenotype) [11,34–36]. Several independent mutations in the *RET* at exons 5, 8, 10, 11, 13, 14, 15 and 16, have been established as causative of MEN 2A, MEN 2B and FMTC [8–13].

The majority of families with MEN 2A (more than 90%) present point mutations in the *RET* protooncogene (*missense* type), involving codons located in the extracellular domain of the receptor: 609, 611, 618 and 620 (exon 10) and 634 (exon 11). The most frequent mutations are located in codon 634, occurring in more than 60% of all genetically identified MTC [11,13,32,37]. Codon 634 mutations have been associated with the presence of pheochromocytoma and hyperparathyroidism [38], and rarely with CLA [39]. Nevertheless, there are a variety of phenotypic expressions in families with the same *RET* mutation [9,11,12,35,38]. Puñales *et al*. observed that the genotype C634R (TGC/Cys → CGC/Arg, exon 11) presented significantly more distant metastases at diagnosis than groups C634W (Cys/TGC → Trp/TGG, exon 11) and C634Y (Cys/TGC → Tyr/TAC, exon 11), thus suggesting that a change of specific amino acids may modify the natural development of the disease [36]. A recent study evaluated the *RET* C634W-specific neoplastic risk and age-related penetrance profiles and found that penetrance is high for MTC (52% by age 30, 83% by age 50 and 98% by age 70) and pheochromocytoma (20% by age 30, 67% by age 50 and 92% by age 70) [40]. In contrast to well-defined risk profiles for carriers of the codon 634 mutations, consensual clinical guidelines for *RET* exon 10 mutation are still being defined. Risk profiles and penetrance estimations in MEN 2A caused by germline *RET* exon 10 mutations were recently analyzed by Frank-Raue *et al*. (2011) in a large multicenter study that included 340 subjects from 103 families. The authors observed that mutations affect mainly the cysteine codons 609, 611, 618 and 620 and 50% penetrance was achieved by the age of 36 years for MTC, by 68 years for pheochromocytoma, and by 82 years for HPT [41]. These data may facilitate risk assessment and genetic counseling for MTC.

MEN 2B occurs, in approximately 95% of the cases, through a specific M918T mutation (exon 16), resulting in the structural change of the intracellular domain of the RET protein. In about 2–3% of patients with MEN 2B, the genotype A883F (GCT → TTT, exon 15) can be found [42,43]. In addition, a double mutation V804M/Y806C at codon 804 (Val/GTG → Met/ATG, exon 14) and 806 (Tyr/TAC → Cys/TGC) in the same allele was described in a patient with MEN 2B. Patients presenting with “atypical” MEN 2B harboring the germline double point mutation in codons 804 and 904 (V804M and S904C) were also reported [44,45]. Mutations in codons 883 and 918 are associated with younger age of MTC onset and higher risk of metastases and disease-specific mortality [11,31,46].

In FMTC, germline mutations are distributed throughout the *RET* gene. Approximately 86–88% of FMTC families have mutations in one of the 5 cysteines in the extracellular domain of the *RET* gene in exons 10 (codons 609, 611, 618, 620) and exon 11 (codon 634) [12,47]. Substitutions in the intracellular domain of *RET* in exon 13 (codon 768, 790, 791), in exon 14 (codon 804 and 844) and in exon 15 (codon 891) are less frequent. Interestingly, the most frequent mutation in MEN 2A, C634R, has not been described in FMTC families [11,47–50].

Based on genotype-phenotype correlation studies, the American Thyroid Association (ATA) developed recommendations for age of prophylactic thyroidectomy in asymptomatic *RET* mutation carriers. The different mutations are classified into four risk categories according to the aggressiveness of the disease (A < B < C < D). Children with mutations associated with MEN 2B phenotype (ATA level D risk) are at highest risk for early development of MTC and should have thyroidectomy as soon as possible, preferably within the first year of life. Patients with codon 634 mutations (ATA level C risk) are also at higher risk for development of MTC at early ages and the prophylactic total thyroidectomy should be carried out before 5 years of age. In patients with ATA level A and B *RET* mutations (codons 768, 790, 791, 804, 891 and 609, 611, 618, 620, 630 respectively), the risk for MTC is moderate and the prophylactic total thyroidectomy may be delayed beyond the age of 5 years if there is a less aggressive MTC family history, a normal basal stimulated serum calcitonin and normal neck ultrasound [51].

## 5. Sporadic Medullary Thyroid Carcinoma

Sporadic MTC generally presents as a unifocal tumor or a palpable cervical lymph node. Diagnosis tends to be late, generally in the fifth or sixth decade of life [52]. Lymph node metastases are detected in at least 50% of these patients, while distant metastases occur in ~20% of cases [53,54]. A minority of patients with MTC present systemic manifestations which include diarrhea, flushing, or painful bone metastases [6].

### Somatic *RET* Mutations and Disease Phenotype

In sporadic MTC, somatic mutation in exon 16 of the *RET* (M918T) has been identified in 50–80% of the patients [14–17]. Somatic mutations in codons 618, 603, 634, 768, 804 and 883 and partial deletion of the *RET* gene have been identified in few tumors [53,54]. The presence of a somatic *RET* mutation correlates with a worse outcome for MTC patients, not only because of the higher probability of persistent disease, but also because of a lower survival rate in a long-term follow up [53,54].

The somatic *RET* mutations (exons 10, 11 e 16) have also been described in other endocrine tumors. Mutations associated with MEN 2A (codon 634 and 631) and 2B (codon 918) phenotype are also found in about 15–20% of sporadic pheochromocytomas [55,56].

## 6. Role of *RET* Polymorphisms in Medullary Thyroid Carcinoma

Since the identification of the *RET* proto-oncogene as the susceptibility gene for hereditary MTC, major advances have been observed in studies concerning the pathogenesis of MTC and associated neoplasias [9,13]. However, certain aspects of the disease, such as the clinical heterogeneity observed in individuals who have the same mutation, are not yet well understood [36,57,58]. As to sporadic MTC, the picture is slightly more obscure, since *RET* somatic mutations are not found in all cases [15,21,46,59] and appear not to occur uniformly among the different subpopulations of cells in the tumor [14,15,60]. In recent years, several authors have investigated whether the presence of variant sequences or polymorphisms could be associated with susceptibility for the development or progression of MTC. These studies have described an increased prevalence of the *RET* polymorphisms G691S (exon 11, rs1799939), L769L (exon 13, rs1800861), S836S (exon 14, rs1800862), and S904S (exon 15, rs1800863) in individuals with hereditary or sporadic MTC when compared with the population [17,57,60–62]. Below, we will discuss the main aspects related to these polymorphisms and susceptibility to MTC development.

### 6.1. *RET* G691S and S904S Polymorphisms

The non synonymous variant G691S (Gly/GGT → Ser/AGT) has been associated with developing sporadic MTC in two larger studies [61,63]. In an Italian population it was demonstrated that the frequency of G691S polymorphism was greater in patients with sporadic MTC compared to the controls (27.8% *vs*. 18.9% *P* = 0.029). Moreover, the authors observed that G691S polymorphism presents a positive significant co-segregation with S904S (SerTCC → SerTCG) polymorphism [61]. Additionally, Cebrian *et al*. (2005), have demonstrated a 1.5 to 2.5 -fold increase in the relative risk for the development of MTC in patients who presented polymorphisms in exons 11 (G691S), 15 (S904S) and 19 (STOP+388bp) [63]. These two studies postulated, through a functional assessment of *RET* transcription and splicing, that G691S could be the functional variant, but the results were inconclusive [61,63]. Fugazzola *et al*. (2008) also tested the functional activity of the *RET* G691S variant and show that the *RET*9-G691S protein was overrepresented when compared to *RET*9-WT. However, no transforming activity was observed [64].

Robledo *et al*., in 2003, also described a strong co-segregation between polymorphisms G691S and S904S, reporting a strong linkage disequilibrium between these polymorphisms. Additionally, it was also demonstrated that haplotype G691S/S904S, in homozygosis, was more prevalent in patients with MEN 2A compared to the control group, suggesting a role as a gene with low penetrance for this variant. Furthermore, the authors observed that this variant (G691S/S904S) could modify the age of onset of MTC patients [57]. However, these data were not replicated in a large sample of European population [65].

Although several studies have found an association between G691S/S904S polymorphisms and MTC, some authors did not observe a difference in the frequency of this variant between MTC patients and the general population [17,66–68]. Wohllk *et al.* analyzed 50 Chilean patients with sporadic tumors and 50 controls of similar ethnic origins, and showed a similar frequency of the *RET* G691S/S904S variants for cases and controls [69]. More recently, these negative results were replicated in Polish, Brazilian and Indian populations [17,67,68].

### 6.2. *RET* L769L Polymorphism

In 2001, a study conducted by Wiench *et al*. reported that patients with sporadic MTC and under the age of 30 years presented a higher frequency of the variant L769L (LeuCTT → LeuCTG) allele than those diagnosed between 31–45 years (36% *vs.* 15%, *P* = 0.04), suggesting that this polymorphism was associated with younger age at diagnosis. However, the absence of a control group diminished the relevance of this observation [58]. Interestingly, Magalhães *et al*. (2004) observed that a patient harboring a V804M mutation, classically associated with late-onset and lower aggressiveness MTC, associated with the L769L polymorphism presented clinically evident MTC at 32 years of age, in contrast to her asymptomatic mother, who had only the V804M mutation and had MTC diagnosed by fine-needle aspiration biopsy at 60 years of age. The authors suggest that polymorphism L769L of *RET* proto-oncogene may be related to younger age at the onset of disease [70].

An association between the presence of L769L polymorphism and F769Y mutation was reported in FMTC patients for Baumgartner-Parzer *et al*. In this study, the authors deduced from pedigree analyses that the F791Y mutation and L769L polymorphism are located on the same allele and speculated whether the presence of this polymorphism could predispose the respective allele for the occurrence of a F791Y *de novo* mutation or would modulate the disease phenotype [66].

More recently, the presence of polymorphism L769L in the *RET* gene was associated with predisposition to the development of sporadic MTC and also younger age at onset of MTC in carriers of the homozygous polymorphic variant L769L. The authors also demonstrated that this variant modifies the structure of mRNA and could lead to changes in kinase activity and/or specificity of the protein [68].

Conversely, other studies did not show an association between the L769L polymorphism and MTC [60,61,63,69]. Berard *et al*. analyzed the presence of the L769L polymorphism in patients with sporadic MTC and controls, and found no difference in the distribution of these polymorphisms between the groups analyzed [71]. Accordingly, Siqueira *et al*. did not observe the influence of neutral *RET* L769L variants on clinical and oncological features in individuals with hereditary or sporadic MTC [17]. Recently, a study performed in Indian patients also failed to demonstrate a difference in the frequency of this allele in MTC patients and control group [67].

### 6.3. *RET* S836S Polymorphism

Gimm *et al*., in 1999, identified an association between the *RET* polymorphisms S836S (SerAGC → SerAGT) and sporadic MTC. The authors reported a higher frequency of the variant allele in the group with MTC compared with the control group (9.0 *vs.* 3.7% *P* = 0.03) [60]. These findings were confirmed in a Spanish population [72]

A recent study investigated the influence of the neutral *RET* S836S variants on the clinical presentation of hereditary or sporadic MTC in a large cohort of Brazilian patients. The variant S836S was associated with the early onset of the disease and a higher risk for the development of lymph node and distant metastases (*P* = 0.002 and *P* = 0.001, respectively) in patients with hereditary or sporadic MTC [17].

Other association studies, however, have failed to show differences as to the presence of S836S polymorphisms between patients with sporadic MTC and controls [61,63,68,69]. Wiench *et al*. in a Polish population and Berard *et al*. in French patients observed a similar frequency of the *RET* S836S variants for cases and controls [58,71]. Similar data were found in other populations [61,63,68,69]. Study performed in India did not observe significant differences in the frequency of this polymorphic allele in the patients and control group. Interestingly, the prevalence of the *RET* polymorphisms in the Indian population was significantly higher than those observed in Germans, Italians, French, Spanish and Hungarians (*P* > 0.002) [67].

### 6.4. Other *RET* Variants

Besides the variants already mentioned, other polymorphisms have also been associated with MTC. A study showed higher frequency of intron 14 (IVS14–24; rs2472737) polymorphism in the group with elevated serum calcitonin concentrations (*P* = 0.016) and in patients with sporadic MTC (*P* < 0.001), when compared with the control group with normal calcitonin levels. However, further studies are necessary to characterize a potential role of this *RET* sequence variant in the development of sporadic MTC [66].

Recently, two other variants of *RET* were identified (IVS1–126 G > T; rs2565206) and (IVS8+82 A > G; rs3026750 and 85–86 insC; rs3482797), and associated with phenotypic variability in patients with mutation G533C. In this study, the authors found an association between variant IVS1–126 G > T and age at diagnosis of MTC. On the other hand, variant (IVS8+82 A > G; InsC 85–86) was associated with the presence of lymph node metastases at the time of diagnosis. Analyses in silico suggest that this variant may induce abnormal splicing, postulating that variant (IVS8+82 A > G; 85–86 InsC) could interrupt and/or create an exonic splicing site, thus leading to the synthesis of an altered protein [73]. In another study, a polymorphism in exon 2 (GCG → GCA), which encodes an alanine (A45A), occurred at a lower frequency among the cases of MTC and, according to the authors, it could confer a protective allele against the development of MTC [63].

Taken together, these data point to a potential influence of *RET* variants in the development and progression of MTC. Tables 1 and 2 summarize the main findings of the studies on the role of *RET* polymorphisms in MTC.

### 6.5. Possible Mechanisms of Action for *RET* Polymorphism in Medullary Thyroid Carcinoma

So far it is not known how polymorphisms exert their effects on the development or progression of MTC and the mechanistic explanation is still speculative. A quantitative study of *RET* mRNA levels in tumor tissues of individuals with MTC did not show a difference in the expression in patients with and without G691S/S904S polymorphism [61]. The S836S polymorphism failed to affect DNA-protein binding, transcript stability, or RNA splicing and editing [74]. Other hypothesis is that bases exchange in the DNA molecule could interrupt and/or create a splicing site, leading to the synthesis of an altered protein, or else, that the modified nucleotide is in a state of linkage disequilibrium with an as yet unknown functional variant [60,73]. It has also been proposed a specific effect of G691S polymorphism on RET dimerization on MEN 2A patients harboring the 634 mutation [57]. Potential changes on mRNA structure due to the presence of *RET* polymorphisms have also been evaluated. The simplest prediction of mRNA structure is a prediction of thermodynamic stable structure, MFE (minimal free energy) structure. Bioinformatics analysis showed that differences in MFE between wild types and mutants are <5% in the case of polymorphisms S904S and S836S and mutations Y791F and C634R. No effect on MFE was visible also in the combination of C634R and L769L polymorphism. However, the difference was noticeable in the case of exon 13. The L769L variant reduces the energy of the wild type by 17% and the mutant Y791F by 7%, leading the authors to conclude that the L769L polymorphism reduces the MFE of small *RET* mRNA [68]. Finally, in silico analysis revealed that the IVS1–126 G>T genetic variant creates a new binding site for NFAT transcription factor (nuclear factor of activated T-cells) [75]. The NFAT family of proteins has been found to be involved in cell cycle regulation, cell differentiation, cell survival, angiogenesis, tumor cell invasion, and metastasis [76], which may explain the association of this variant with disease progression [73].

## 7. Conclusion

In summary, since the recognition of the *RET* proto-oncogene as the susceptibility gene for hereditary MTC several decades ago, advances have taken place in understanding pathogenesis of MTC and associated neoplasias. Nevertheless, certain aspects of the disease, such as the clinical heterogeneity seen in individuals harboring the same mutation have not yet been well understood. Polymorphisms in the *RET* gene are commonly associated with MTC and may partially explain the large clinical heterogeneity observed in MEN 2A patients. An entire set of data obtained from clinical studies indicates a potential role of *RET* polymorphisms in the development of sporadic MTC. However, in contrast, several others failed to demonstrate any association between these *RET* variants and MTC development or disease progression. Although differences in ethnic background or methodological flaws might be potential causes for the different results described, the mechanism underlying the positive associations is still lacking which stimulates further controversy. Since the contribution of a single variant to a disease is determined by the prevalence of the implicated allele and the magnitude of the association with the condition, the results summarized here might indicate the need for large multicenter studies to confirm or rule out a role of these variants as a cause or modifying agent in this rare disease.

## Figures and Tables

**Table 1:** Role of the RET variants in hereditary medullary thyroid cancer.

*RET* variant	Author	Cases	Controls	*P*	Frequency (cases *vs.* controls)	Genotyping platform	Conclusion	Population
**G691S (rs1799939)**	Robledo (2003)	198	653	0.037	–	sequencing	Associated with the presence of MTC in younger individuals.	Spanish
	Lesueur (2006)	384	–	–	–	Taqman	N/A	European
	Tamanah a (2009)	77 a	100	0.048	0; 4	RFLP	Underrepresented in G533C-arriers.	Brazilian
	Sharma b (2011)	51	50	NS	49; 48	sequencing	N/A	Indian

**L769L (rs1800861)**	Sharma b (2011)	51	50	NS	45; 58	sequencing	N/A	Indian

**S836S (rs1800862)**	Tamanah a (2009)	77 a	100	0.008	16.9; 4	RFLP	Over-represented in G533C-carriers.	Brazilian
	Siqueira (2010)	88	–	–	7.95; –	RFLP	Associated with early onset and increased risk for metastatic disease.	Brazilian
	Sharma b (2011)	51	50	NS	25; 22	sequencing	N/A	Indian

**S904S (rs1800863)**	Lesueur (2006)	384	–	–	–	Taqman	N/A	European
	Sharma b (2011)	51	50	NS	25; 22	sequencing	N/A	Indian
	Tamanah a (2009)	77 a	100	0.048	0; 4	RFLP	Underrepresented in G533C-carriers.	Brazilian

**IVS1–126 G>T (rs2565206)**	Tamanah a (2009)	77 a	100	0.002	1.3; 0	RFLP	Associated with younger age at diagnosis.	Brazilian

**IVS8+82 A>G; 85–86 insC (rs3026750)**	Tamanah a (2009)	77 a	–	0·019	–	RFLP	Associated with lymph node metastases. Could induce abnormal splicing.	Brazilian

a Study performed in patients with *RET* G533C mutation;

b The study included hereditary and sporadic MTC patients; N/A: no association was found.

**Table 2:** Role of the *RET* variants in sporadic medullary thyroid cancer.

*RET* variant	Author	Cases	Controls	*P*	Frequency (cases *vs.* controls)	Genotyping platform	Conclusion	Population
**G691S/S904S (rs1799939)/(rs1800863)**	Elisei (2004)	106	106	0.029	27.8; 18.8	RFLP	Higher frequency in MTC patients. Does not influence RET mRNA expression	European
	Cebrian a (2005)	120	528	0.004	27; 18	TaqMan	Associated with higher risk for development of MTC. Does not affect the splicing of RET	British
	Wohllk (2005)	50	50	NS	25; 25	sequencing	N/A	Chilean

**L769L (rs1800861)**	Wiench (2001)	116 b	–	0.04 b	36; 15	sequencing	Associated with the presence of MTC in younger individuals	Polish
	Sromek (2010)	217	420	0.039^c^	48.3; 39.5 c	Sequencing	Associated with the presence of MTC in younger individuals (in homozygosis). Could influence RET mRNA structure	Polish
	Berard (2004)	184	174	NS	22.3; 25.9	sequencing	N/A	French
	Wohllk (2005)	50	50	NS	24; 23	sequencing	N/A	Chilean

**S836S (rs1800862)**	Gimm (1999)	50	70	0.03	9; 3.7	RFLP	More frequent in MTC patients	German- American
	Ruiz (2001)	32	250	0.04	9.3; 3.6	RFLP	Associated with higher risk for development of MTC	Spanish
	Siqueira (2010)	81	80	0.01	10.5; 3.2	RFLP	Associated with early onset and increased risk for metastatic disease	Brazilian
	Berard (2004)	184	174	NS	6.5; 5.2	sequencing	N/A	French
	Wohllk (2005)	50	50	NS	6; 1	sequencing	N/A	Chilean

**S904S (rs1800863)**	Wohllk (2005)	50	50	NS	27; 28	sequencing	N/A	Chilean
	Cebrian (2005)	125	528	0.005	26.4; 15.5	TaqMan	Associated with higher risk for development of MTC	British

**STOP+388pb G>A (rs3026782**	Cebrian (2005)	123	522	0.005	26.4; 15.5	TaqMan	Associated with higher risk for development of MTC	British

**A45A G>A (rs1800858)**	Cebrian (2005)	126	525	0.04	21; 27.9	TaqMan	Suggest protective effect	British

a Study did not confirm the previously described association between G691S and S904S;

b The comparison was performed between patients aged below and above 30 years;

c Frequency of heterozygous change L769L.N/A: no association was found.
